# Psychological distress, body image, and nutritional status during hospitalization for gynecological cancer surgery: a prospective observational study

**DOI:** 10.1007/s00520-025-09862-3

**Published:** 2025-08-21

**Authors:** Letizia Lafuenti, Francesca Ciccarone, Rebecca De Paola, Svetlana Sicco, Livia Sani, Daniela Belella, Luca Liberati, Daniele Ferrarese, Valentina Massaroni, Anna Fagotti, Daniela Pia Rosaria Chieffo

**Affiliations:** 1 Clinical Psychology Unit, Health Management, Fondazione Policlinico Universitario Agostino Gemelli IRCCS, Largo Agostino Gemelli 1, Rome, 00168 Italy; 2 Gynecologic Oncology Unit, Department of Woman and Child Health and Public Health, Fondazione Policlinico Universitario A. Gemelli IRCCS, Rome, 00168 Italy; 3 Department of Health Science and Public Health, Faculty of Medicine and Surgery, Catholic University of the Sacred Heart, 00168 Rome, Italy; 4 Medicine and Surgery Faculty, Catholic University of the Sacred Heart, Rome, 00168 Italy

**Keywords:** Gynecological cancer, Psychological distress, Body image, Orthorexia nervosa, Nutritional status, Supportive care

## Abstract

**Background:**

Hospitalization for gynecological cancer surgery represents a critical window for assessing and addressing psychological and nutritional vulnerabilities. This prospective observational study investigated changes in emotional distress, anxiety, depression, body-image dissatisfaction, orthorexic tendencies, and nutritional status from admission to discharge, and explored associations between psychological and nutritional variables.

**Methods:**

A total of 220 women hospitalized for surgical treatment of gynecological cancer were enrolled, with 181 (82.3%) completing both baseline (T0) and discharge (T1) assessments. Psychological outcomes were evaluated using the Distress Thermometer (DT), Hospital Anxiety and Depression Scale (HADS), Body-Image Scale (BIS), and Teruel Orthorexia Scale (TOS). Nutritional status was assessed through the Mini Nutritional Assessment (MNA). Changes between T0 and T1 were analyzed using paired *t*-tests. Pearson’s correlations examined associations between psychological and nutritional variables. A multivariable logistic regression identified predictors of clinically relevant distress (DT ≥ 4) at discharge.

**Results:**

Significant improvements were observed in anxiety (*p* < 0.001), depression (*p* < 0.001), emotional distress (*p* < 0.001), and orthorexic tendencies (*p* < 0.001) between admission and discharge. Conversely, body-image dissatisfaction increased significantly (*p* < 0.001). Nutritional risk remained high throughout hospitalization, with no statistically significant change (*p* = 0.221). Higher body-image dissatisfaction at admission predicted a greater likelihood of clinically relevant distress at discharge (*p* = 0.003).

**Conclusions:**

Hospitalization offers a pivotal opportunity to identify and address emotional and nutritional needs in women with gynecological cancers. Integrated, multidisciplinary supportive care models targeting both psychological and nutritional vulnerabilities are crucial to promote holistic recovery during and beyond the surgical course.

## Introduction

Gynecological cancers, including cervical, ovarian, uterine, vaginal, and vulvar malignancies, represent a substantial health burden for women worldwide, accounting for nearly 40% of all female cancer cases [1]. Although advances in early detection and treatment have improved survival rates, the physical and psychological sequelae associated with these diseases and their treatments remain profound [2].

Surgical procedures are fundamental to the management of diseases; however, they frequently result in body-image disturbances, heightened emotional vulnerability, and psychological distress, which may include anxiety and depression [3, 4]. These psychological challenges can adversely impact treatment adherence and overall quality of life [5]. The consequences of surgical interventions extend beyond their physical effects, significantly influencing women’s embodied experiences, particularly concerning perceptions of femininity, attractiveness, and bodily integrity [6–8]. In the context of gynecological oncology, the body serves as both a site of healing and a focal point of loss, often leading to a dissonant or disrupted body image [9, 10]. Body-image dissatisfaction, characterized by a negative perception of an individual’s physical appearance and the functionality of the body, has been linked to increased emotional distress, diminished coping abilities, and a decreased quality of life [11, 12]. Moreover, body-image distress has been identified as a notable predictor of postoperative psychological outcomes and treatment adherence. It is increasingly being integrated into patient-reported outcome measures (PROMs) within oncological research [13].

In addition to the psychological burden, patients with gynecological cancers frequently encounter nutritional challenges [14–16]. Reduced appetite, taste alterations, and an increased risk of malnutrition are common, particularly during hospitalization and chemotherapy regimens [17–19]. Malnutrition has been associated with poorer clinical outcomes, delayed recovery, and elevated healthcare costs [18]. Moreover, the psychological impact of a cancer diagnosis may foster maladaptive eating behaviors, such as orthorexia nervosa—a pathological fixation on healthy eating—that can further compromise psychological well-being and quality of life [19, 20].

While multidisciplinary interventions combining psychological support and nutritional counseling have shown promise in oncology care [21–23], limited evidence exists regarding the dynamic interplay between psychological and nutritional factors during hospitalization for gynecological cancer surgery. In recent years, psychological models addressing the embodied aspects of the cancer experience have been developed. Notably, Sebri et al. [24] introduced an intervention grounded in the concept of body compassion, which aims to cultivate a more accepting and compassionate relationship with the body following treatment. This approach, rooted in third-wave cognitive-behavioral frameworks, offers a targeted strategy for diminishing feelings of shame, enhancing body image, and promoting psychological adaptation. Furthermore, other intervention models focusing on body-image restructuring, narrative re-elaboration, and expressive therapies have shown preliminary efficacy in oncological settings [25–27]. However, the implementation of these interventions within inpatient perioperative protocols remains infrequent, and their integration into routine care continues to be limited.

There exists a significant gap in empirical research that has systematically examined the co-evolution of body image, emotional distress, and nutritional vulnerability during the hospitalization process. Additionally, the potential role of body-image dissatisfaction as a risk factor for postoperative distress warrants further investigation. Exploring these variables within the acute surgical context may yield crucial insights into patients’ adaptation trajectories and inform the development of more comprehensive supportive care models. Given the interplay between body-related vulnerability and nutritional-emotional imbalances, the timely identification of risk profiles during hospitalization may present a critical opportunity for personalized intervention strategies.

Hospitalization represents a pivotal period for assessing changes in emotional and nutritional status. Understanding the evolution of distress, anxiety, depression, body-image concerns, and nutritional risk during hospitalization may help identify patients at heightened risk for adverse outcomes and inform the development of integrated, patient-centered supportive care strategies.

### Objectives

This prospective observational study aimed to investigate the following:

- Primary objective
  - Assess the prevalence of clinically significant psychological distress at the time of discharge and identify preoperative psychological and nutritional factors that may predict this distress.
- Secondary objectives
  - Describe within-hospital changes (admission–discharge) in psychological outcomes (distress, anxiety, depression, body-image dissatisfaction) and nutritional status (malnutrition risk, orthorexic tendencies).
  - Examine cross-sectional correlations between psychological and nutritional variables at admission and at discharge.

### Hypotheses

We hypothesized that several preoperative variables—body-image dissatisfaction, anxiety, depression, orthorexic tendencies, and malnutrition risk—would each be positively associated with clinically relevant distress at discharge. Based on prior literature, we anticipated that body-image dissatisfaction might emerge as a particularly strong predictor within this set. As secondary expectations, we anticipated declines in mean distress, anxiety, and depression by discharge (perioperative stabilization), minimal short-term change in malnutrition risk, and reductions in orthorexic tendencies within the structured inpatient environment.

## Methods

### Study aim and design

This prospective observational study was conducted at the Complex Operative Unit of Gynecological Oncology at Fondazione Policlinico Universitario Agostino Gemelli IRCCS in Rome, Italy. The Unit operates within a multidisciplinary framework integrating gynecologists, oncologists, psycho-oncologists, and nutritionists. Data collection occurred during standard clinical practice between October 2023 and June 2024.

### Compliance with ethical standards

The study was conducted by the principles of the Declaration of Helsinki. Ethical approval was obtained from the Lazio Area 3 Territorial Ethics Committee (Protocol ID 5913). Written informed consent was obtained from all participants. Data were anonymized and securely stored according to institutional policies. Psychological and nutritional assessments were conducted at hospital admission (T0) and discharge (T1) as part of standard clinical care. Data were collected by the clinical psychologists and the ward nutritionist while patients were awaiting admission. All questionnaires were completed autonomously by the participants, with an average completion time of approximately 15 min. Each questionnaire was subsequently anonymized through the assignment of a unique code, accessible exclusively to the first author, by the guidelines of the local ethics committee, to ensure participant confidentiality and data protection. Patients had access to routine psychological support and nutritional advice, delivered following institutional guidelines.

### Patient population and recruitment

Eligible participants were adult women (≥ 18 years) hospitalized for surgical treatment of gynecological malignancies and able to provide informed consent. Exclusion criteria included major psychiatric comorbidities, inability to complete assessments, explicit refusal to participate, or language barriers. Major psychiatric comorbidities were identified through review of medical records and pre-admission clinical interviews and included acute psychosis, severe mood disorders with psychotic features, or significant cognitive impairment secondary to psychiatric illness. Such conditions were considered likely to compromise the patient’s ability to engage in the assessment process, maintain adequate insight, or provide valid self-report data. Language barriers were characterized as a total inability to comprehend or communicate in Italian, which would impede the understanding of questionnaire items and potentially compromise the accuracy of responses, even with researcher support. In such instances, the engagement of professional interpreters was not practicable within the confines of the study protocol, and the involvement of family members as proxies was precluded due to inadequate proficiency in Italian. Consequently, only patients with sufficient proficiency in Italian to understand both the instructions and the content of the questionnaire were included in the study.

Participants were recruited consecutively at the time of hospital admission by a clinical psychologist and a trained research assistant. Eligible patients were approached during the pre-operative phase and invited to participate after a brief explanation of the study. A total of 220 patients were initially enrolled; 181 patients (82.3%) completed both assessments (T0 and T1). Losses to follow-up (17.7%) were primarily due to early weekend discharges and logistical limitations. A descriptive analysis was conducted to compare baseline demographic and clinical variables between completers and non-completers, evaluating potential attrition bias. The results revealed no significant differences between the two groups.

### Measures

Psychological outcomes were assessed using the following validated instruments:

- Hospital Anxiety and Depression Scale (HADS) [28]: A 14-item self-report questionnaire measuring symptoms of anxiety (HADS-A) and depression (HADS-D). Scores range from 0 to 21 per subscale. Cutoffs are the following: 0–7 = normal, 8–10 = mild, 11–14 = moderate, and 15–21 = severe distress. The Italian validated version was used in this study [29]. Cronbach’s alphas were 0.88 for HADS-A and 0.84 for HADS-D.
- Distress Thermometer (DT) [30]. The DT is a single-item visual analog scale (0–10) assessing emotional distress. We adopted the commonly accepted threshold of ≥ 4 to indicate clinically relevant distress, as supported by multiple validation studies and meta-analytic evidence showing that this cutoff provides optimal sensitivity and specificity for detecting psychological distress in oncology populations, including both inpatients and outpatients [31–36]. Data were collected using the Italian validated version [37]. The DT is widely used in oncology and shows good test–retest reliability; Cronbach’s alpha is not applicable given its single-item structure.
- Body-Image Scale (BIS) [38]: A 10-item measure assessing cancer-related body-image disturbances. Higher scores reflect greater dissatisfaction; there are no established clinical cutoffs. The version of the scale validated for use in Italy was adopted [39]. Cronbach’s alpha for the BIS in our sample was 0.90.
- Teruel Orthorexia Scale (TOS) [40]: A 17-item scale evaluating orthorexic tendencies, divided into Healthy Orthorexia (HeOr) and Orthorexia Nervosa (OrNe) subscales. Higher scores indicate greater orthorexic attitudes; there are no validated clinical cutoffs. The Italian validated version was used in this study [41]. Cronbach’s alphas were 0.82 for OrNe and 0.79 for HeOr.

Nutritional outcome was assessed with the following:

- Nutritional outcome was assessed with the Mini Nutritional Assessment (MNA) [42], a 30-point screening tool evaluating nutritional status. Cutoffs are the following: ≥ 24 = normal nutrition, 17–23.5 = at risk of malnutrition, and < 17 = malnutrition. This study employed the Italian version of the instrument [43]. Cronbach’s alpha for the MNA was 0.80. The MNA is recommended by the Italian Ministry of Health’s national guidelines on nutritional pathways in oncology, which endorse it as a validated instrument with high sensitivity (100%) and specificity (92%) in identifying individuals at risk of malnutrition [44].

Demographic and clinical variables included age, body mass index (BMI), cancer site, surgical approach (laparoscopy vs. laparotomy), employment status, geographical origin, history of eating disorders (EDs), and food intolerances. The history of EDs was assessed during the pre-assessment clinical interview, conducted jointly by a clinical psychologist and the ward nutritionist, as part of the standard perioperative psychosocial and nutritional evaluation. This assessment followed institutional practice for inpatient psycho-oncology consultations, which routinely include questions on sleep–wake patterns, dietary habits, and any past or current diagnosis of an ED. When available, information was cross-checked against the patient’s electronic medical record to corroborate self-reported history. Participants were reassured that all information would remain confidential and would not influence their medical care, to encourage accurate disclosure. BMI was recorded upon hospital admission as part of the standard clinical assessment, typically by the ward dietitian or, when applicable, by the medical or nursing staff. While BMI is also a component of the MNA, in this study, it was collected as a separate demographic and clinical variable to enable exploratory analyses of potential correlations with other study measures. Importantly, BMI was not intended to serve as the sole indicator of nutritional status, as the MNA was the primary nutritional screening instrument.

### Outcomes

The primary outcome was psychological distress at T1, operationalized as a DT score ≥ 4. Secondary outcomes included changes in anxiety (HADS-A), depression (HADS-D), body-image dissatisfaction (BIS), orthorexic tendencies (TOS), and nutritional status (MNA) between T0 and T1.

The minimum sample size was calculated a priori to estimate the proportion of patients experiencing clinically relevant distress (DT ≥ 4) at discharge, assuming a prevalence of 50% (maximizing variance), a 95% confidence level, and a 7% margin of error. Based on these parameters, the required sample size was 196 participants. To account for potential attrition, an additional 12% was planned, resulting in a recruitment target of 220 participants, in line with institutional experience from previous inpatient studies. The actual attrition rate was higher (17.7%), yielding 181 completers. Although this number was slightly below the original minimum requirement, the loss in precision was marginal (margin of error ≈ 7.3%), and statistical power remained adequate for the primary analysis.

### Data analysis

All analyses were conducted using Jamovi [45]. Descriptive statistics were used to summarize demographic, clinical, psychological, and nutritional characteristics. Changes in continuous psychological and nutritional measures between hospital admission (T0) and discharge (T1) were analyzed using paired *t*-tests.

Pearson’s correlation coefficients were calculated to explore associations between psychological and nutritional variables at T0 and T1.

A multivariable logistic regression analysis was performed to identify independent predictors of clinically relevant emotional distress (distress thermometer ≥ 4) at discharge, adjusting for demographic, clinical, and psychological factors.

Missing data were addressed through complete case analysis. The potential for attrition bias was examined by comparing key baseline variables between participants who completed the study and those who did not, indicating no statistically significant differences. Additionally, attrition bias was further assessed through a descriptive comparison of the available baseline characteristics of completers and non-completers.

All statistical tests were two-tailed, and statistical significance was set at *p* < 0.05.

## Study results

### Sample characteristics

As presented in Table 1, a total of 220 women diagnosed with gynecological cancers were enrolled upon hospital admission (T0). Among these, 181 patients (82.3%) completed the follow-up assessment at discharge (T1), while 39 participants (17.7%) were lost to follow-up. The primary reasons for loss to follow-up included early weekend discharges and logistical challenges associated with administering questionnaires. The attrition rate for this study was approximately 18%.

**Table 1:** Demographic and clinical characteristics of the sample at admission (T0)

Variable	*n* (%) or mean (SD)
Cancer site	
Ovary	74 (40.9)
Uterus	30 (16.6)
Endometrium	18 (9.9)
Vulva	18 (9.9)
Awaiting diagnosis	41 (22.7)
Type of intervention	
Laparotomy	49 (27.1)
Laparoscopy	132 (72.9)
Age (years)	48.8 ± 14.2
≤ 39	134 (74.0)
> 39	47 (26.0)
BMI	
Normal	122 (67.4)
Pathological (underweight/overweight/obese)	59 (32.6)
History of eating disorders	
Yes	10 (5.0)
No	171 (95.0)
Food intolerances	
Yes	51 (28.2)
No	130 (71.8)
Risk of malnutrition at T0 (MNA)	
At risk	163 (90.1)
Not at risk	18 (9.9)
Geographic origin	
Northern Italy	2 (1.1)
Central Italy	114 (63.0)
Southern Italy	52 (28.7)
Foreign country	13 (7.2)
Work situation	
Employed	122 (67.4)
Unemployed	17 (9.4)
Student	8 (4.4)
Housewife	15 (8.3)
18 (10.5)	19 (10.5)

*BMI* body mass index, *MNA* Mini Nutritional Assessment, *n* number, *SD* standard deviation

Comparative analyses between completers and non-completers were limited by extensive missing data in the dropout group, particularly concerning psychological and nutritional variables. However, based on available clinical information (e.g., type of cancer and surgical approach), no substantial differences were identified between groups.

### Demographic and clinical characteristics

The final sample comprised 181 women who completed both T0 and T1 assessments. The mean age of participants was 48.8 years (SD = 14.2), with the majority aged ≤ 39 years (74%; *n* = 134). Ovarian cancer was the most frequent diagnosis (40.9%; *n* = 74), followed by endometrial and cervical cancers.

Regarding surgical approach, 72.9% (*n* = 132) underwent laparoscopic surgery, reflecting the clinical preference for minimally invasive procedures. Most participants (63%; *n* = 114) originated from central Italy and were actively employed (67.4%; *n* = 122). Normal BMI values were reported in 67.4% (*n* = 122) of the sample.

Eating disorder history was absent in 95% (*n* = 171) of patients, and 71.8% (*n* = 130) reported no known food intolerances. Baseline nutritional screening revealed that 90.1% (*n* = 163) were classified as “at risk of malnutrition” according to the Mini Nutritional Assessment (MNA) at T0.

Detailed demographic and clinical characteristics are summarized in Table 1.

## Psychological and nutritional outcomes

Between admission (T0) and discharge (T1), significant changes were observed across several psychological and nutritional parameters. Anxiety symptoms, as measured by the Hospital Anxiety and Depression Scale (HADS-A), decreased from a mean of 7.96 (SD = 4.23) at T0 to 4.62 (SD = 3.56) at T1 (*p* < 0.001). Similarly, depression scores (HADS-D) significantly declined from 4.19 (SD = 2.75) to 2.48 (SD = 2.16) (*p* < 0.001). Emotional distress, assessed through the Distress Thermometer (DT), also showed a marked reduction, with mean scores decreasing from 6.35 (SD = 2.49) to 3.30 (SD = 2.49) (*p* < 0.001).

Conversely, body-image concerns appeared to intensify during hospitalization. The mean score on the Body-Image Scale (BIS) increased from 6.62 (SD = 6.91) at admission to 8.74 (SD = 7.38) at discharge (*p* < 0.001), suggesting a heightened self-consciousness related to surgical and physical changes.

Orthorexic tendencies, as assessed by the Teruel Orthorexia Scale (TOS), demonstrated significant improvements. Healthy Orthorexia (HeOr) scores decreased from 6.00 (SD = 4.27) to 4.39 (SD = 3.79) (*p* < 0.001), while Orthorexia Nervosa (OrNe) scores declined from 18.87 (SD = 4.07) to 17.29 (SD = 4.75) (*p* < 0.001), indicating reduced rigidity and pathological fixation on healthy eating.

Regarding nutritional status, 90.1% (*n* = 163) of participants were categorized as “at risk of malnutrition” at T0, compared to 87.8% (*n* = 159) at T1. Although this reduction was not statistically significant (*p* = 0.221), it highlights the persistent vulnerability of patients in the postoperative period.

All changes across psychological and nutritional outcomes are detailed in Table 2.

**Table 2:** Psychological and nutritional outcomes at admission (T0) and discharge (T1)

Variable	T0 Mean (SD) or %	T1 Mean (SD) or %	*p* value
HADS Anxiety	7.96 (4.23)	4.62 (3.56)	< 0.001
HADS Depression	4.19 (2.75)	2.48 (2.16)	< 0.001
Distress Thermometer	6.35 (2.49)	3.30 (2.49)	< 0.001
Body-Image Scale (BIS)	6.62 (6.91)	8.74 (7.38)	< 0.001
TOS Healthy Orthorexia (HeOr)	6.00 (4.27)	4.39 (3.79)	< 0.001
TOS Orthorexia Nervosa (OrNe)	18.87 (4.07)	17.29 (4.75)	< 0.001
Risk of malnutrition (MNA)	90.1%	87.8%	0.221

*HADS* Hospital Anxiety and Depression Scale, *MNA* Mini Nutritional Assessment, *T0* hospital admission, *T1* discharge

## Correlations between psychological and nutritional outcomes

Significant correlations were observed between psychological distress, emotional symptoms, body-image concerns, orthorexic tendencies, and nutritional status at both T0 and T1 assessments.

At admission (T0), anxiety symptoms (HADS-A) showed a positive correlation with body-image dissatisfaction (BIS) (*r* = 0.354, *p* < 0.001), indicating that higher anxiety levels were associated with greater concerns about body image. Similarly, depressive symptoms (HADS-D) correlated positively with BIS scores (*r* = 0.351, *p* < 0.001). Distress levels (DT) also demonstrated a moderate positive correlation with BIS (*r* = 0.229, *p* < 0.001).

Regarding the relationship between psychological and nutritional outcomes, negative correlations were found between nutritional status (MNA) and both anxiety (HADS-A; *r* =  − 0.204, *p* = 0.006) and depression (HADS-D; *r* =  − 0.264, *p* < 0.001), suggesting that worse nutritional status was associated with higher emotional distress.

At discharge (T1), similar patterns persisted. Anxiety (HADS-A) remained positively associated with body-image dissatisfaction (BIS) (*r* = 0.231, *p* = 0.002), and depressive symptoms (HADS-D) correlated strongly with emotional distress (DT) (*r* = 0.426, *p* < 0.001).

A detailed overview of the correlation coefficients and significance levels is provided in Table 3.

**Table 3:** Correlations between psychological and nutritional variables at admission (T0) and discharge (T1)

Variables	*r* Pearson	*p* value
HADS Anxiety and BIS (T0)	0.354	< 0.001
HADS Depression and BIS (T0)	0.351	< 0.001
Distress Thermometer and BIS (T0)	0.229	< 0.001
HADS Anxiety and MNA (T0)	− 0.204	0.006
HADS Depression and MNA (T0)	− 0.264	< 0.001
HADS Anxiety and BIS (T1)	0.231	0.002
HADS Depression and DT (T1)	0.426	< 0.001

*HADS* Hospital Anxiety and Depression Scale, *MNA* Mini Nutritional Assessment, *BIS* Body-Image Scale, *T0* hospital admission, *T1* discharge

## Multivariable analyses

A multivariable logistic regression model including demographic, clinical, psychological, and nutritional factors was conducted to identify predictors of clinically relevant distress (DT ≥ 4) at discharge (T1).

After adjustment, higher body-image dissatisfaction at admission (BIS T0) was significantly associated with a greater likelihood of distress at discharge (OR = 1.10, 95% CI 1.03–1.17, *p* = 0.003).

The presence of a history of disordered eating (DCA) was associated with a reduced probability of distress, although this association reached only borderline significance (OR = 0.17, 95% CI 0.03–1.00, *p* = 0.050).

Other variables, including age, BMI, type of cancer, surgical approach, baseline anxiety (HADS-A), depression (HADS-D), distress (DT), nutritional status (MNA), employment status, and presence of food intolerances, were not significantly associated with distress outcomes (all *p* > 0.05).

Full results of the multivariable model are presented in Table 4.

**Table 4:** Multivariable logistic regression predicting clinically relevant distress (DT ≥ 4) at discharge (T1)

Predictor variable	Odds ratio (OR)	95% confidence interval (CI)	*p* value
Intercept	4.85	0.06–363.85	0.473
Age (continuous)	0.98	0.96–1.01	0.283
BMI (continuous)	0.98	0.91–1.05	0.622
Ovarian cancer (vs. others)	0.78	0.29–2.06	0.616
Laparoscopic approach (vs. laparotomy)	0.95	0.44–2.04	0.888
HADS Anxiety at T0	1.10	0.99–1.22	0.085
HADS Depression at T0	0.93	0.80–1.08	0.349
BIS at T0	1.10	1.03–1.17	**0.003**
DT at T0	1.16	0.99–1.37	0.074
MNA at T0	0.88	0.75–1.04	0.134
Employment status (working)	0.37	0.10–1.34	0.129
Presence of EDs	0.17	0.03–1.00	0.050
Presence of food intolerances	1.34	0.59–3.03	0.480

*HADS* Hospital Anxiety and Depression Scale, *MNA* Mini Nutritional Assessment, *BIS* Body-Image Scale, *DT* distress thermometer, *BMI* body mass index, *EDs* eating disorders, *T0* hospital admission

## Discussion

This prospective observational study explored changes in psychological and nutritional outcomes among women undergoing surgery for gynecological cancers. Between hospital admission and discharge, significant improvements were observed in anxiety, depression, emotional distress, and orthorexic tendencies, while body-image concerns moderately worsened. Although the study design does not allow for causal conclusions, these findings suggest that hospitalization may represent a pivotal period for addressing emotional and nutritional vulnerabilities in this population. These findings are consistent with the existing body of literature that indicates the perioperative period may serve as a psychological inflection point. This period has the potential either to exacerbate vulnerability or to provide an opportunity for stabilization and support [46, 47].

Younger women have reported elevated levels of psychological distress, which aligns with prior research indicating their increased vulnerability following a cancer diagnosis [48]. This trend can be understood in the context of the developmental challenges related to identity, fertility, and body image that are prominent in early adulthood. These aspects may be disproportionately impacted by oncological treatment [49, 50]. A history of disordered eating has been associated with heightened body-image dissatisfaction at both assessed time points, highlighting the necessity for tailored psychological interventions that address pre-existing vulnerabilities [51]. This observation is consistent with research indicating that pre-existing body-related psychopathology may serve as a risk factor for post-treatment body-image distress, even in the absence of overt physical disfigurement [52, 53]. Furthermore, the surgical approach appears to influence outcomes significantly; patients who underwent laparotomy reported more pronounced body-image disturbances than those treated with minimally invasive laparoscopic techniques [54]. This finding underscores the psychological benefits associated with less invasive surgical procedures [55]. Minimally invasive approaches are increasingly recommended, not only for their advantages related to physical recovery but also for their potential to mitigate patients’ subjective experiences of mutilation and identity disruption [56, 57].

Concerning nutritional status, a consistently high proportion of women were classified as “at risk of malnutrition” both at the time of admission and at discharge. These individuals were subjected to regular monitoring, and when clinically indicated, they were referred to the hospital dietitian for nutritional counseling or provided with oral nutritional supplements by the standard perioperative care protocol. However, the lack of a structured or standardized nutritional intervention protocol within the study may explain the absence of significant improvement in overall nutritional risk during hospitalization. This finding is consistent with previous studies that emphasize the vulnerability of patients with gynecological cancers [58]. While there was no statistically significant improvement in nutritional risk during hospitalization, notable decreases in orthorexic tendencies were recorded. This observation may indicate a reduction in rigid dietary behaviors under the supervision of medical professionals, despite the global nutritional status remaining unchanged over the short term [59]. The observed divergence can be attributed to the distinct constructs that are evaluated. Nutritional risk, as quantified by the MNA, encompasses a chronic and multifaceted vulnerability influenced by enduring dietary habits, baseline nutritional status, and metabolic alterations related to disease. These factors are unlikely to experience significant change within the brief duration of hospitalization. In contrast, orthorexic tendencies reflect maladaptive cognitive-behavioral attitudes towards food, which may be more readily influenced by the structured environment of a hospital [60]. Elements such as standardized meal provision, medical oversight, and a temporary alleviation of personal responsibility regarding food choices may facilitate a reduction in rigid eating behaviors during inpatient treatment [61]. Therefore, hospitalization may serve as a corrective environment for disordered eating attitudes, despite not substantially altering the underlying nutritional risk [62].

In our institution, the clinical pathway for nutritional and psychological care during hospitalization adheres to a structured yet adaptable model. Nutritional assessments are conducted by the ward dietitian through unstructured interviews focusing on dietary habits, food preferences, and eating behaviors, along with BMI measurements. For this study, the MNA was systematically integrated into the admission process to provide a standardized and validated evaluation of patients’ nutritional status. Those identified as at risk are closely monitored and may be provided with individualized counseling or oral nutritional supplements. Psychological assessments are typically initiated through a physician’s referral to the psycho-oncology service based on observed concerns. In this study, all participants completed the DT at the time of admission, which facilitated uniform screening across the board. Individuals who scored above the established distress threshold were referred for further evaluation, and brief consultations were offered as necessary. Both nutritional and psychological interventions are tailored to meet the specific needs of each patient, reflecting the standard practices of perioperative care while being enhanced by research-driven screening protocols. It is essential to consider this context when interpreting the findings, as the observed changes transpired within the framework of routine perioperative care, supplemented by the standardized screening procedures specifically implemented for this study.

Psychological and nutritional health are intricately interconnected, as evidenced by the relationships between emotional distress, body-image concerns, and compromised nutritional status [63, 64]. Recent biopsychosocial frameworks have underscored the necessity of integrating nutritional and psychological pathways, demonstrating that malnutrition may intensify inflammation, which subsequently exacerbates mood disorders and cognitive fatigue in cancer patients [65–67]. These findings highlight the complex, bidirectional relationship between physical and emotional domains within cancer care. Furthermore, recent studies indicate that malnutrition not only worsens emotional symptoms but also serves as a predictor of poorer treatment outcomes, thereby reinforcing the imperative for integrated care models [68].

Improvements in emotional distress, anxiety, and depression may, in part, reflect both the natural psychological adjustment that occurs following surgery and hospitalization [69–71] and the supportive measures provided to patients with elevated distress scores. Such patients were routinely referred to the hospital psycho-oncology service for further assessment and, when appropriate, received brief supportive consultations during hospitalization. These interventions were part of standard clinical care, although not standardized within the study protocol, and may have contributed to the observed short-term reductions in distress. At the same time, research indicates that emotional trajectories during the perioperative period among oncological patients can fluctuate independently of specific interventions, as highlighted in systematic reviews [72]. Consequently, any observed changes should be interpreted with caution, as they cannot be exclusively attributed to the supportive care received during hospitalization. Nonetheless, the early postoperative period may present a critical opportunity for targeted psychological intervention, particularly for patients exhibiting preoperative risk factors such as younger age, previous disordered eating, or a poor baseline body image [73–75].

The findings presented highlight the necessity for an integrated, multidisciplinary approach to supportive care that comprehensively addresses both psychological and nutritional needs during hospitalization for gynecological cancers. This multidimensional support should encompass structured psycho-oncological consultations, systematic screening for body image and nutritional issues, as well as ongoing psychoeducational dialogue throughout the clinical pathway [76–80]. The early identification of vulnerable demographics—such as younger women and individuals with pre-existing body-image concerns—is crucial for the effective tailoring of interventions. Recent models advocate for a patient-centered, multidisciplinary approach to oncological care that not only aims at improving clinical outcomes but also emphasizes emotional well-being and nutritional support [71]. Promising interventions include body compassion therapy [24], narrative exposure therapy [81], and expressive body-based therapies [82, 83], which address the complex psychosomatic responses associated with cancer and surgical interventions. The implementation of systematic psychosocial screening and nutritional assessments at critical junctures during hospitalization could enhance the personalization of care and facilitate improved recovery trajectories. This approach is particularly pertinent in the field of gynecological oncology, where the intersections of corporeality, identity, and emotional significance hold substantial symbolic implications [84, 85].

Future research should prioritize longitudinal designs to assess the sustainability of emotional and nutritional changes following hospitalization. Furthermore, it will be essential to formally evaluate patient satisfaction with supportive interventions to guarantee that care aligns with the unique needs and expectations of individuals. The development of co-designed intervention protocols that incorporate patient involvement has the potential to enhance the clinical relevance and adherence to psychosocial and nutritional strategies [86, 87].

## Study limitations

Strengths of the study include the prospective design, the use of validated and standardized instruments for psychological and nutritional assessment, and the application of multivariable analysis to adjust for potential confounders. These elements contribute to the robustness and internal validity of the findings.

However, several limitations must be acknowledged. First, the observational design without a control group precludes causal inferences. Second, an attrition rate of 17.7% should be acknowledged as a limitation. Although comparative analyses did not reveal major demographic or clinical differences between completers and non-completers, attrition bias is unlikely to have significantly impacted the overall findings, although it cannot be entirely excluded. Third, analyses were performed utilizing a complete case approach, which involved excluding participants with missing data on the primary variables. This method is frequently employed, as it presumes that the data are missing completely at random (MCAR). While comparisons indicated no significant differences between participants who completed the study and those who did not, the potential for bias resulting from non-random missingness cannot be entirely dismissed. Fourth, the absence of tumor staging information and the relatively short follow-up period limit the generalizability of the results. Fifth, the assessment of ED history relied primarily on self-report during clinical interview, with partial verification through medical records when available. Although this approach reflects routine perioperative clinical practice, the sensitive nature of EDs may have led to underreporting or incomplete disclosure despite reassurances of confidentiality, potentially underestimating their prevalence in the sample. Sixth, although no formal PROMs were collected regarding satisfaction with psychological or nutritional counseling, informal feedback gathered during clinical encounters indicated a generally high level of patient appreciation toward the supportive care provided. Nevertheless, the lack of systematic evaluation prevents firm conclusions about patient satisfaction and alignment with individual needs. Finally, the predominance of younger patients from Central Italy may limit the applicability of findings to broader and more diverse populations.

## Conclusions

Hospitalization for gynecological cancer surgery represents a critical window to address emotional distress, body-image concerns, and maladaptive eating behaviors. While improvements in psychological outcomes were observed during hospitalization, nutritional risk remained prevalent, highlighting the need for continuous, integrated supportive care. Future studies should aim to confirm these findings over longer follow-up periods, expand to more heterogeneous populations, and incorporate patient-centered evaluations to better guide the development of holistic, multidisciplinary models of care for women facing gynecological malignancies. Given the observational design, these findings should be interpreted with caution, and further controlled studies are warranted to confirm these associations.

## Data Availability

The datasets generated and analyzed during the current study are available from the corresponding author upon reasonable request.
